# Assessing PFAS exposure in Rocky Mountain elk (*Cervus canadensis nelsoni*) populations adjacent to the former Rocky Flats nuclear site: A preliminary analysis

**DOI:** 10.1371/journal.pone.0334258

**Published:** 2025-12-01

**Authors:** David Lucas, Kelsey E. Schreiber, Joseph M. Halseth, Meghan C. W. Williams, Philip Sorensen, Benjamin R. Kraft, Megan E. Klosterman, Thomas J. Ronning, Wayne Soong, Monique A. Rivera

**Affiliations:** 1 Colorado Front Range National Wildlife Refuge Complex, United States of America Fish and Wildlife Service, Commerce City, Colorado, United States of America; 2 Toxicology and Environmental Epidemiology Office, Colorado Department of Public Health and Environment, Denver, Colorado, United States of America; 3 Colorado Parks and Wildlife, Colorado Department of Natural Resources, Denver, Colorado, United States of America; Indian Institute of Technology Delhi, INDIA

## Abstract

The Rocky Flats National Wildlife Refuge is located west of Denver, Colorado, USA along the central Front Range of the Rocky Mountains. The wildlife refuge property includes a former U.S. Department of Energy nuclear weapons facility, hereafter “the former Rocky Flats site.” Owing to the storage and use of aqueous film forming foam (AFFF) at the on-site fire department, industrial areas of the former Rocky Flats site has detectable concentrations of per- and polyfluoroalkyl substances (PFAS) in groundwater (range perfluorooctanesulfonic acid [PFOS]: not detected [ND] [<0.75]–350 ng/L, perfluorooctanoic acid [PFOA]: ND [<0.55]–160 ng/L) and surface water (range PFOS: 1.9–18 ng/L, PFOA: ND [<0.55]–13 ng/L). AFFF is a known source of PFOA and PFOS contamination in the environment. PFAS are a class of thousands of human-made chemicals considered ubiquitous in the environment globally. Exposure to these chemicals can negatively impact the health of both humans and wildlife. In March 2023, we used a draft version of the U.S. Environmental Protection Agency Method 1633 to analyze liver and muscle tissues from 17 Rocky Mountain elk (*Cervus canadensis nelsoni*) to detect the presence of PFAS. We collected samples from 15 elk residing at the former Rocky Flats site and 2 elk at locations with no known PFAS contamination (i.e., control locations). Neither PFOA nor PFOS was detected in elk muscle tissues collected at the former Rocky Flats site or the control locations. The average method detection limits for PFOA and PFOS in muscle tissues were 0.166 ng/g and 0.154 ng/g respectively. PFOS was detected within 100% of the liver tissues harvested from elk at the former Rocky Flats site and both control locations. The average PFOS concentrations in liver tissues collected at the former Rocky Flats site and control locations were 16.23 ± 4.40 ng/g (maximum concentration 34.20 ng/g) and 8.75 ± 3.02 ng/g (maximum concentration 9.40 ng/g), respectively. To the best of our knowledge, this is the first study examining PFAS concentrations in elk. Although we were unable to draw conclusions owing to the relatively small sample size, PFOS concentrations in liver tissues collected at the former Rocky Flats site were low, consistent with those detected in other species of wildlife studied in the United States with known PFAS contamination.

## Introduction

The Rocky Flats National Wildlife Refuge is located west of Denver, Colorado, USA along the central Front Range of the Rocky Mountains. The wildlife refuge was established in 2007 to preserve native prairie ecosystems and provide habitat for migratory and resident wildlife including the federally threatened Preble’s meadow jumping mouse (*Zapus hudsonius preblei*). Prior to becoming a wildlife refuge, the U.S. Department of Energy (USDoE) used portions of the property to build weapons components for the United States’ nuclear weapons arsenal, hereafter referred to as “the former Rocky Flats site.” Most of the former Rocky Flats site has remained undisturbed since its acquisition by the federal government, creating favorable conditions for fish and wildlife conservation. A subherd of Rocky Mountain elk (*Cervus canadensis nelsoni*) inhabits the area and continues to grow in numbers. As of February 2023, this subherd of elk comprised at least 360 individuals [1] increasing to at least 405 by February 2025 [2].

In recent years, the USDoE performed document searches and confirmed that the on-site fire department at the former Rocky Flats site used aqueous film forming foam (AFFF), which is commonly known as firefighting foam. AFFF is a well-documented source of per- and polyfluoroalkyl substances (PFAS) contamination in the environment. Polyflouroalkyl substances (PFAS) are a large class of thousands of synthetic chemicals, often termed “forever chemicals,” and are recognized as extremely persistent and considered ubiquitous in the environment globally [3–5]. PFAS exposure is associated with a range of adverse health effects in humans and wildlife. In humans, the primary pathways of PFAS exposure include drinking water and dietary intake [6]. Although the U.S. Environmental Protection Agency (EPA) has finalized enforceable limits in public drinking water systems in 2024 [7], a consensus has yet to be reached regarding safe levels of dietary exposure to PFAS. Nonetheless, a growing body of *in vitro*, animal, and epidemiological studies has demonstrated the negative effects of PFAS exposure at environmentally relevant levels. Available evidence most strongly highlights the relationship between PFAS exposure and altered immune function, kidney and liver disease, lipid dysregulation, low infant birth weight, and increased cancer risk [8–11]. Health impacts may vary by the amount, duration, and frequency of exposure, as well as by factors such as age, sex, and genetic predisposition [12].

Perfluorooctanesulfonic acid (PFOS) is a chemical of particular interest, and its production has been restricted or banned in several countries. In the environment, PFOS behaves like a persistent organic pollutant (POP), although it appears to lack certain important characteristics of more familiar POPs. For example, most POPs accumulate in lipids, whereas PFOS binds to proteins [13]. This necessitates distinct techniques for monitoring PFOS in biota and has implications for recommendations provided to the public regarding the safe consumption of contaminated game and fish. Similar to other POPs, PFOS is characterized by its persistence and bioaccumulation. Houde et al. provided an initial review of PFAS in biota in 2006, and further summarized the substances in aquatic ecosystems in 2011, demonstrating that PFOS remains as the predominant PFAS found in all species, tissues, and locations analyzed worldwide [14,15]. Typically, PFOS concentrations in wildlife are higher in more populated and urbanized areas than in more remote areas [5]. However, there are no regulatory standards established for perfluorooctanoic acid (PFOA) or PFOS in biota and no standards regulating the consumption of livestock or wildlife by humans.

In 2019, the USDoE initiated a limited water sampling program for two PFAS, PFOA and PFOS, at the former Rocky Flats site. PFOA and PFOS were detected in both ground and surface water at locations within former industrial areas. The highest levels of PFOA (120 ng/L) and PFOS (310 ng/L) were detected in the wells located behind and downgradient of the former fire station. Average PFOA and PFOS levels across the two surface water sampling locations at the downstream end of the former industrial area were substantially lower (mean PFOA = 4.25 ng/L maximum 13 ng/L; mean PFOS = 5.75 ng/L, maximum 18 ng/L) [16]. In 2021, the USDoE expanded the number of sampling locations and began employing a different analytical method to detect 28 analytes. In 2023, the USDoE initiated another study to compare its current analytical method with a draft version of the U.S. EPA Method 1633, with the results between 2019 and 2023 revealing the presence of PFAS in groundwater (range PFOS: not detected [ND] [< 0.75]–350 ng/L, PFOA: ND [< 0.55]–160 ng/L) and surface water (range PFOS: 1.9–18 ng/L, PFOA: ND [< 0.55]–13 ng/L) [17].

In 2019, Colorado Parks and Wildlife and the U.S. Fish and Wildlife Service (USFWS) initiated a five-year project to assess and better understand the distribution of elk along the central Front Range of Colorado. The central Front Range is a corridor of the Rocky Mountains, which comprises both highly urban and rural areas. This interface creates opportunities for large elk herds to find refugia in local parks and open spaces within and adjacent to urban areas. This project deployed 40 global positioning system (GPS) collars on female elk across various subherds within the central Front Range population to delineate habitat use and spatial distribution, identify migration corridors and potential highway crossings, and inform future management strategies, including hunting, at both local and herd-wide scales [18]. A few considerations in determining an appropriate management strategy include the potential for older individuals to carry disease (e.g., chronic wasting disease) and the potential contamination of elk tissue with environmental pollutants.

In 2023, the USFWS culled 15 elk at the former Rocky Flats site to test for chronic wasting disease; these elk were also sampled for PFAS. As controls, two elk were sampled from remote areas in Colorado. Liver and muscle tissues of elk were analyzed in a contracted laboratory using a draft analytical method for liquid chromatography tandem mass spectrometry designed to analyze 40 PFAS chemicals including PFOA and PFOS (Table 1). The objectives of this study were to support the development of a future elk management plan; determine whether the prevalence of chronic wasting disease in this subherd of elk is similar to that in other surrounding subherds of elk; and detect the presence or absence of PFOA and/or PFOS in elk.

**Table 1:** Names, Abbreviations, and CAS Registry Numbers for PFAS chemicals included in the U.S. EPA Method 1633 with the unqualified detection frequency located in elk muscle and liver tissues at both the former Rocky Flats site and control locations, Colorado, USA, 2023.

Target Analyte Name	Abbreviation	CARSN	Unqualified Laboratory Frequency of Detection	
			Muscle	Liver
**Perfluoroalkyl carboxylic acids**				
Perfluorobutanoic acid	PFBA	375-22-4	0.0%	0.0%
Perfluoropentanoic acid	PFPeA	2706-90-3	0.0%	0.0%
Perfluorohexanoic acid	PFHxA	307-24-4	0.0%	0.0%
Perfluoroheptanoic acid	PFHpA	375-85-9	0.0%	0.0%
Perfluorooctanoic acid	PFOA	335-67-1	0.0%	0.0%
Perfluorononanoic acid	PFNA	375-95-1	0.0%	0.0%
Perfluorodecanoic acid	PFDA	335-76-2	0.0%	0.0%
Perfluoroundecanoic acid	PFUnA	2058-94-8	0.0%	0.0%
Perfluorododecanoic acid	PFDoA	307-55-1	0.0%	0.0%
Perfluorotridecanoic acid	PFTrDA	72629-94-8	0.0%	0.0%
Perfluorotetradecanoic acid	PFTeDA	376-06-7	0.0%	0.0%
**Perfluoroalkyl sulfonic acids**				
Perfluorobutanesulfonic acid	PFBS	375-73-5	0.0%	0.0%
Perfluoropentanesulfonic acid	PFPeS	2706-91-4	0.0%	0.0%
Perfluorohexanesulfonic acid	PFHxS	355-46-4	0.0%	0.0%
Perfluoroheptanesulfonic acid	PFHpS	375-92-8	0.0%	0.0%
Perfluorooctanesulfonic acid	PFOS	1763-23-1	0.0%	100.0%
Perfluorononanesulfonic acid	PFNS	68259-12-1	0.0%	0.0%
Perfluorodecanesulfonic acid	PFDS	335-77-3	0.0%	0.0%
Perfluorododecanesulfonic acid	PFDoS	79780-39-5	0.0%	0.0%
**Fluorotelomer sulfonic acids**				
1*H*,1*H*,2*H*,2*H*-Perfluorohexane sulfonic acid	4:2FTS	757124-72-4	0.0%	0.0%
1*H*,1*H*,2*H*,2*H*-Perfluorooctane sulfonic acid	6:2FTS	27619-97-2	11.8%	26.5%
1*H*,1*H*,2*H*,2*H*-Perfluorodecane sulfonic acid	8:2FTS	39108-34-4	0.0%	0.0%
**Perfluorooctane sulfonamides**				
Perfluorooctanesulfonamide	PFOSA	754-91-6	0.0%	0.0%
N-methyl perfluorooctanesulfonamide	NMeFOSA	31506-32-8	0.0%	0.0%
N-ethyl perfluorooctanesulfonamide	NEtFOSA	4151-50-2	0.0%	0.0%
**Perfluorooctane sulfonamidoacetic acids**				
N-methyl perfluorooctanesulfonamidoacetic acid	NMeFOSAA	2355-31-9	0.0%	0.0%
N-ethyl perfluorooctanesulfonamidoacetic acid	NEtFOSAA	2991-50-6	0.0%	0.0%
**Perfluorooctane sulfonamide ethanols**				
N-methyl perfluorooctanesulfonamidoethanol	NMeFOSE	24448-09-7	0.0%	0.0%
N-ethyl perfluorooctanesulfonamidoethanol	NEtFOSE	1691-99-2	0.0%	0.0%
**Per- and Polyfluoroether carboxylic acids**				
Hexafluoropropylene oxide dimer acid	HFPO-DA	13252-13-6	0.0%	0.0%
4,8-Dioxa-3*H*-perfluorononanoic acid	ADONA	919005-14-4	0.0%	0.0%
Perfluoro-3-methoxypropanoic acid	PFMPA	377-73-1	0.0%	0.0%
Perfluoro-4-methoxybutanoic acid	PFMBA	863090-89-5	0.0%	0.0%
Nonafluoro-3,6-dioxaheptanoic acid	NFDHA	151772-58-6	0.0%	0.0%
**Ether sulfonic acids**				
9-Chlorohexadecafluoro-3-oxanonane-1-sulfonic acid	9Cl-PF3ONS	756426-58-1	0.0%	0.0%
11-Chloroeicosafluoro-3-oxaundecane-1-sulfonic acid	11Cl-PF3OUdS	763051-92-9	0.0%	0.0%
Perfluoro(2-ethoxyethane)sulfonic acid	PFEESA	113507-82-7	0.0%	0.0%
**Fluorotelomer carboxylic acids**				
3-Perfluoropropyl propanoic acid	3:3FTCA	356-02-5	0.0%	0.0%
2*H*,2*H*,3*H*,3*H*-Perfluorooctanoic acid	5:3FTCA	914637-49-3	0.0%	0.0%
3-Perfluoroheptyl propanoic acid	7:3FTCA	812-70-4	0.0%	0.0%

## Materials and Methods

### Field collection

To determine the presence of chronic wasting disease, 15 elk were culled at the former Rocky Flats site in March 2023. All animal procedures were conducted in accordance with the guidelines and protocols of the USFWS Animal Welfare Program involving vertebrate animals in national wildlife refuges, including the 2016 Guidelines of the American Society of Mammologists for the use of wild animals in research and education [19], AVMA Guidelines for the Euthanasia of Animals (2020 Edition) [20], and the humaneness of gunshot placement to the brain and cervical vertebrae for disease sampling activities [21]. The animals were euthanized by gunshot using all-copper ammunition. After shooting, each animal was quickly assessed to ensure that the euthanasia was humane and effective.

**Fig 1:**
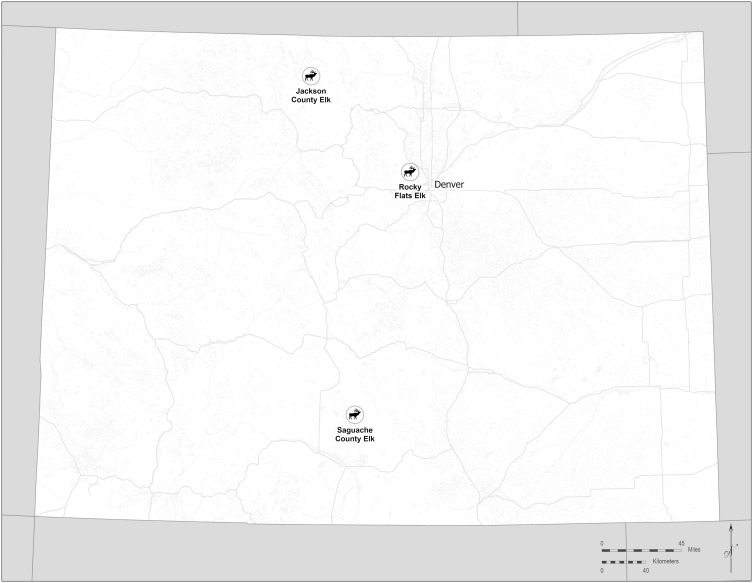
Former Rocky Flats site and control locations used for collection of elk samples, Colorado, USA, 2023. The map was created using ArcGIS Pro 3.2.3 with State boundaries from US Census files (https://www.census.gov/geographies/mapping-files/time-series/geo/carto-boundary-file.html), city locations from CDPHE Open Data (https://data-cdphe.opendata.arcgis.com/maps/CDPHE::colorado-city-point-locations) and sampling locations created by Megan E. Klosterman.

At that time, USFWS staff harvested liver and muscle tissues from the carcasses. Control tissue samples were obtained from animals at the Arapaho National Wildlife Refuge (Jackson County, Colorado, USA) and at the Baca National Wildlife Refuge (Saguache County, Colorado, USA) (Fig 1). Demographic information, including sex, body condition, pregnancy status, and age at tooth replacement and wear, was collected from all culled animals [22]. Where applicable, fetal age was also estimated [23]. Duplicate tissue samples were collected at a 1:10 ratio to confirm the accuracy of the analytical methods.

Given the numerous potential sources of PFAS cross-contamination, the following techniques were utilized to reduce the risk of cross-contamination during sampling. Staff were divided into three groups, and at each step, measures to reduce the potential for cross-contamination were increased. The first group was responsible for locating, culling and transporting the animals to a maintenance facility. The second group operated outside the maintenance facility and was responsible for performing a celiotomy and harvesting the muscles and organs. The third group operated within the facility and was responsible for tissue dissection and sample management. Different cutting and storage utensils were employed by each group and were not shared across the groups. Thorough decontamination process and replacement of cutting blades were performed between each animal.

Personal safety is a priority, and the safety of staff should not be compromised to prevent PFAS cross-contamination. Cool (−3° C) and damp conditions existed during sampling. The staff involved in cutting were provided with oversized 100% cotton lab coats (Dr. James® Cotton Lab Coat, 100% Cotton, Multiple Pockets, Classic Fit). Prior to sampling, lab coats were washed once with detergent and four times without detergent. Staff wore powder-free nitrile gloves throughout the sample collection process (Elara Everfit® 3G Nitrile Disposable Gloves, 3 mil, Black, Powder Free, Food Safe, Non-Latex). The gloves were changed as required, and at a minimum, changed between animals. To collect muscle and organs, quick-change stainless-steel blades (Havalon® Baracuta-Blaze) were used. During dissection, #24 carbon-steel scalpel blades and stainless-steel handles were used in concert with stainless-steel dissection trays and bowls. Individual samples were placed in PFAS-free bags provided by General Engineering Laboratories (GEL) in Charleston, South Carolina, USA.

### Sample collection

Before the sampling period, GEL provided PFAS-free sample bags, adhesive sample labels, and PFAS-free water.

The deceased animals were delivered by vehicle outside of the maintenance facility and placed on the asphalt parking lot. A tag with a unique animal identifier was placed on the left ear. A data sheet was created by visually inspecting the animal’s overall condition. The staff noted the sex and age of animals. The animals were opened using a standard stainless-steel knife. Organ and muscle tissues were collected using a quick-change knife stainless-steel blade and placed in a stainless-steel bowl for transport inside the building for dissection.

A dissection team collected two 30-gram samples of liver tissue (central lobe) and two 30-gram samples of muscle tissue (from the “ribeye” or “tenderloin” portion of the animal). Tissue samples were placed in individual PFAS-free bags with a unique sample identification number and the date and time of collection. All samples from each animal were then placed into a larger Ziploc® baggie and placed in a cooler with cold packs for the remainder of the sampling period.

Between animals, attention was paid to decontamination and avoiding cross-contamination. All surface areas, utensils, and other items were cleaned between animals using Alconox® and PFAS-free water. All staff members changed gloves. All the materials used were removed from the area for proper disposal.

Both equipment and environmental blanks were collected to confirm the absence of cross-contamination during sampling. Equipment blanks consisting of one of each type of cutting blade that were placed in 500 mL of PFAS-free water for 24 hours. Environmental blanks were placed within the maintenance facility adjacent to the dissection area and consisted of 500 mL of PFAS-free water in a bowl. Environmental blanks were collected during the sampling period.

During collection, tissues were stored in a cooler with cold packs. Thereafter, the tissues were then transferred and stored in a secure, sealed freezer (−10° C) until they were shipped for analysis.

### PFAS analyses

The samples were packaged on ice and promptly shipped overnight to GEL for laboratory analyses. Samples were analyzed by GEL using a draft version of the U.S. EPA Method 1633 for the extraction and analysis of PFAS using liquid chromatography tandem mass spectrometry (LC-MS/MS; GL-OA-E-076D REV#1) [24]. This method yields results for 40 analytes.

### Statistical analyses

Basic data analyses were performed using R software (version 4.5.1) [25]. All data are expressed as mean ± standard error of the mean (SEM). An ordinary least squares regression was performed for PFOS concentrations in elk as a function of age, sex, and their interaction [25] comparing trends among male and female elk relative to age by estimating individual slopes and 95% confidence intervals using the emmeans package in R [26]. A *p*-value <0.05 was deemed statistically significant and reported.

## Results

PFOA was not detected in elk tissues collected at the former Rocky Flats site or in samples collected from the control locations. For the selected method, the average method detection limits for PFOA were 0.166 ng/g and 2.150 ng/g in muscle and liver tissues, respectively.

PFOS was not detected in the muscle tissues of elk at any location. For the selected method, the average method detection limit for PFOS in muscle tissues was 0.154 ng/g. PFOS was detected in the liver tissues of elk at the former Rocky Flats site and both control locations. The average PFOS concentration in liver tissues collected at the former Rocky Flats site was 16.23 ± 4.40 ng/g, reaching a maximum concentration of 34.20 ng/g (Table 2). In contrast, the average PFOS concentration in liver tissues collected at control locations was 8.75 ± 3.02 ng/g, reaching a maximum concentration of 9.40 ng/g (Table 3).

**Table 2:** Concentrations of PFOA and PFOS in liver and muscle tissues of the 15 elk sampled at the former Rocky Flats site, 2023.

		PFOA (ng/g)		PFOS (ng/g)	
Sex	Age (years)	Liver	Muscle	Liver	Muscle
**Female**	**<1**	ND (<0.157)	ND (<0.156)	8.8	ND (<0.145)
	**<1**	ND (<3.33)	ND (<0.154)	20.7	ND (<0.143)
	**4**	ND (<3.03)	ND (<0.165)	24.7	ND (<0.153)
	**5**	ND (<0.160)	ND (<0.154)	12.6	ND (<0.143)
	**6**	ND (<2.78)	ND (<0.163)	14.0	ND (<0.152)
	**8**	ND (<0.164)	ND (<0.164) ND (<2.02) ^a^	11.7	ND (<0.152) ND (<0.152) ^a^
	**10**	ND (<0.148)	ND (<0.162)	11.2	ND (<0.150)
	**17**	ND (<0.160)	ND (<0.155)	5.7	ND (<0.144)
**Male**	**<1**	ND (<2.78)	ND (<0.164) ND (<0.158) ^a^	13.4	ND (<0.152) ND (<0.147) ^a^
	**2**	ND (<3.33)	ND (<0.165)	12.1	ND (<0.153)
	**3**	ND (<2.78)	ND (<0.136)	21.6	ND (<0.126)
	**3**	ND (<3.03) ND (<2.22) ^a^	ND (<0.152)	7.3 7.5 ^a^	ND (<0.141)
	**5**	ND (<2.56) ND (<3.03) ^a^	ND (<0.149)	19.4 19.2 ^a^	ND (<0.139)
	**7**	ND (<3.70)	ND (<0.157)	26.0	ND (<0.146)
	**7**	ND (<3.03)	ND (<0.159)	34.2	ND (<0.148)

^a^This table includes duplicate results. Field duplicates and corresponding sample results were used to calculate precision as the relative percent difference (RPD). All the sample pairs met the desired criteria.

**Table 3:** Concentrations of PFOA and PFOS in liver and muscle tissues of elk at the two control locations in rural Jackson and Saguache Counties, Colorado, 2023.

		PFOA (ng/g)		PFOS (ng/g)	
Sex	Age (years)	Liver	Muscle	Liver	Muscle
**Female**	**4**	ND (<3.03)	ND (< 0.164)	9.4	ND (<0.152)
	**7**	ND (<2.38) ND (<2.08) ^a^	ND (< 0.306) ND (< 0.280) ^a^	8.1 7.3 ^a^	ND (<0.284) ND (<0.260) ^a^

^a^This table includes duplicate results. Field duplicates and corresponding sample results were used to calculate precision as the relative percent difference (RPD). All the sample pairs met the desired criteria.

Although this study focused on PFOA and PFOS (both long-chain C_8_-PFAS substances), 6:2 FTS (a short-chain C_6_-PFAS substance) was also detected in elk tissues at the former Rocky Flats site and one control location during the method analysis (S1 and S2 Tables). Notably, 6.2 FTS is a known alternative for PFOS in AFFF and various industrial applications [27,28].

**Fig 2:**
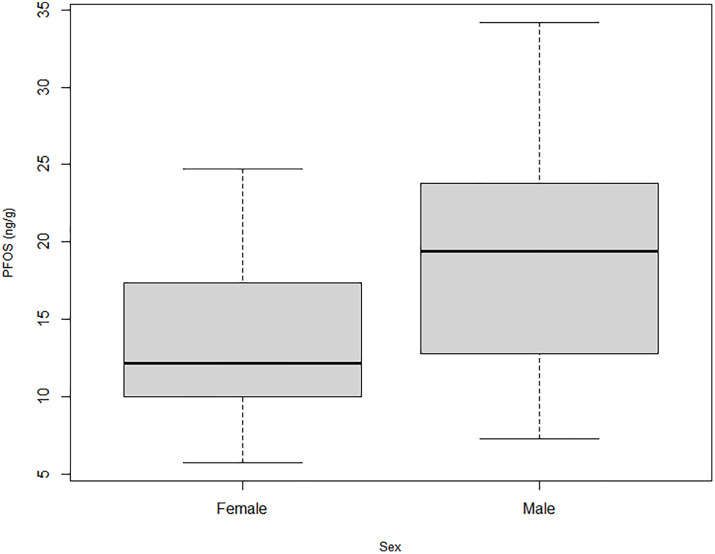
Concentrations of PFOS in the liver of elk sampled at the former Rocky Flats site, 2023.

**Fig 3:**
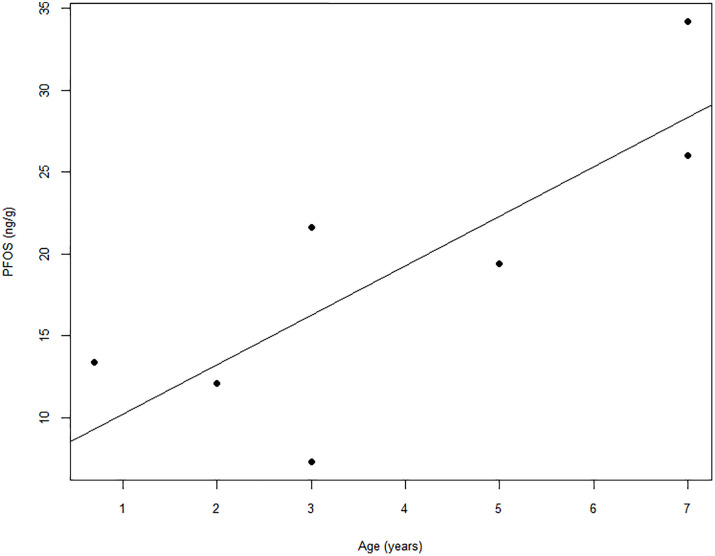
PFOS concentrations in the liver tissue of male elk sampled at the former Rocky Flats site, 2023.

PFOS concentrations in liver tissues of male elk (*x* = 19.14 ± 8.47) were higher than those in female elk (*x* = 13.67 ± 5.19) (Fig 2). In male elk, age and PFOS concentrations in liver tissues moderately correlated (r(5) = 0.57290; *p* = 0.032475) (Fig 3).

All breeding-age females sampled at the former Rocky Flats site were pregnant. Both control animals were female but were not pregnant. Liver and muscle samples were collected from two fetuses at the former Rocky Flats site. Both fetuses were estimated to be more than 123 days old. PFOA was not detected in the liver or muscle tissues of either fetus. PFOS was not detected in the muscle tissue of either fetus. However, a PFOS concentration of 10.8 ng/g was detected in the liver of one fetus, compared with a concentration of 11.2 ng/g in the liver tissue of the mother. Although PFOS was not detected in the second fetus sampled, a concentration of 11.7 ng/g was detected in the liver tissue of the mother (Table 4).

**Table 4:** Concentrations of PFOA and PFOS in liver and muscle tissues of two “family pairs” at the former Rocky Flats site, 2023.

		PFOA (ng/g)		PFOS (ng/g)	
		Liver	Muscle	Liver	Muscle
**Pair 1**	**Mother**	ND (<0.148)	ND (<0.162)	11.2	ND (<0.150)
	**Fetus**	ND (<2.08)	ND (<0.142)	10.8	ND (<0.132)
**Pair 2**	**Mother**	ND (<0.164)	ND (<0.164) ND (<2.02) ^a^	11.7	ND (<0.152) ND (<0.152) ^a^
	**Fetus**	ND (<0.162)	ND (<0.163)	ND (<0.150)	ND (<0.152)

^a^This table includes duplicate results. Field duplicates and corresponding sample results were used to calculate precision as the relative percent difference (RPD). All the sample pairs met the desired criteria.

### PFAS were not detected in the equipment and environmental blanks

Overall, the sample data met GEL’s acceptance criteria for initial calibration, continuous calibration, instrument controls and process controls. Specific to PFOA and PFOS, the relative percentage difference (RPD) of the matrix spike and its duplicate matrix spike was outside the acceptable limits. However, a percentage change between relatively low analyte concentrations can produce a large RPD. In this study, the PFOS concentration in the original matrix spike was 16.6 ng/g and 39.0 ng/g in its duplicate.

Duplicate pairs of elk tissues analyzed by GEL revealed a strong correlation, increasing confidence in the reported values for PFOS in liver tissues.

## Discussion

### Comparison with other ungulate PFAS studies

To the best of our knowledge, this is the first study to analyze PFAS concentrations in elk. However, other state agencies have sampled white-tailed deer (*Odocoileua virginianus*) from PFAS-impacted sites and analyzed the muscle and liver tissues to determine their PFAS concentrations (Table 5). In 2018 and 2020, the Michigan Department of Health and Human Services (MDHHS) analyzed PFAS in 64 white-tailed deer sampled from lands immediately adjacent to a U.S. Air Force base with known PFAS contamination [29]. In 2021, the Maine Department of Inland Fisheries and Wildlife analyzed PFAS in eight white-tailed deer sampled near farms with high levels of PFAS contamination in the soil and surface water [30]. In 2020, the Wisconsin Department of Natural Resources analyzed PFAS in 20 white-tailed deer collected near an AFFF manufacturing facility, with elevated levels of PFAS known to be present in nearby surface waters [31].

**Table 5:** Concentrations of PFOS in surface water and ungulate liver and muscle tissues at four United States locations, 2018–2023.

Location (species) (year)	N ^a^	PFOS Concentration		
		Surface Water (ng/L)	Liver (ng/g)	Muscle (ng/g)
Colorado, Rocky Flats (elk) (2023)	15	ND (<0.75)–18	5.7–34.2	ND (<0.146)
Maine, Fairfield (white-tailed deer) (2021)	8	128–7,330	5.5–890	1.4–43.5
Michigan, Oscoda (white-tailed deer) (2018, 2020)	64	83–1,410	3.46–2,970	0.34–82.6
Wisconsin, Marinette (white-tailed deer) (2020)	20	ND (<1.8)–10	3.83–92	ND (<1.09)–2.67

^a^Represents the number of animals from which tissue samples were collected.

Consistent with the findings of the present study, PFOS was the most frequently detected PFAS, with the highest concentrations reported in white-tailed deer tissues. Similar patterns of tissue distribution have been documented, with higher PFOS concentrations in the liver than in the muscle in both elk (this study) and white-tailed deer (previous studies) (Table 5). These results are were consistent with the PFAS distribution patterns reported for various livestock and wild game species [32,33]. The liver reportedly accumulates high levels of PFAS because plasma albumin is synthesized in the liver, and PFAS binds preferentially to plasma albumin proteins [34].

Notably, our findings for sex and age were inconsistent with those of previous studies. Considering sex, our finding that liver tissues harvested from male elk contained higher levels of PFOS than liver tissues of females is consistent with that observed in Michigan white-tailed deer [29], but inconsistent with a previous study examining PFAS in roe deer (*Capreolus capreolus*) from Italy, which reported higher PFAS in female livers than in males [33], and with findings from Wisconsin, which reported no difference in PFOS liver concentrations between the sexes of white-tailed deer [35]. Likewise, our finding that PFOS levels increased with age was inconsistent with findings from Wisconsin, which did not report age-related trends in liver PFOS concentrations [35]. Nevertheless, our findings were consistent with those reported by Draghi et al. in roe deer, where the authors hypothesized that an increase in PFOS with age could be attributed to reduced liver function and slower metabolism in older animals [33].

### Surface water as an exposure source

Surface water consumption is presumed to be the primary PFAS exposure pathway for ungulates, and this assumption can be evaluated using the data from this study and other states. At the former Rocky Flats site, PFOS concentrations in the surface water have reportedly ranged from below the detection limit to 18 ng/L [16]. In contrast, substantially higher levels have been reported elsewhere. In Michigan, marshes adjacent to the Air Force base contained PFOS at concentrations between 83 and 1,410 ng/L [29]. In Maine, surface waters near contaminated agricultural fields contained PFOS concentrations ranging between 128 and 7,330 ng/L [30]. In Wisconsin, the surface water near an AFFF manufacturing facility showed PFOS concentrations similar to those at the former Rocky Flats site, ranging from below the detection limit to 10 ng/L [36].

**Fig 4:**
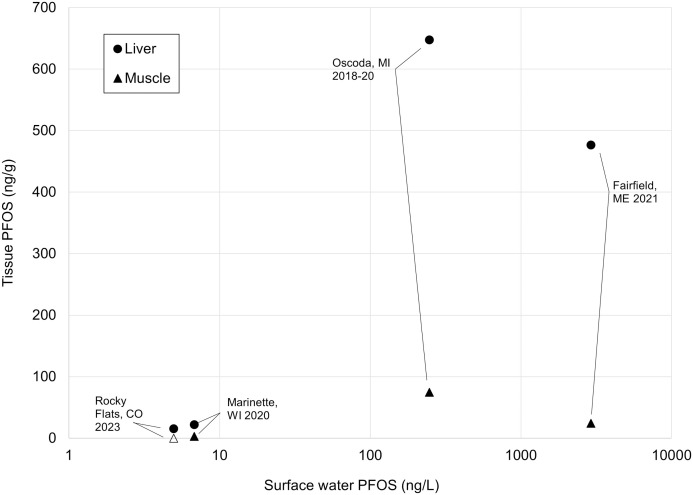
Average PFOS concentrations in surface waters (ng/L) and ungulate liver and muscle tissues (ng/g) sampled at four locations. Circles indicate liver PFOS concentrations and triangles represent muscle tissue concentrations. An open triangle at the former Rocky Flats site reflects that PFOS was not detected in muscle.

The average PFOS concentrations in the liver and muscle of elk (this study) and white-tailed deer (previous studies) positively correlate with those in nearby surface waters (Table 5, Fig 4). At the former Rocky Flats and Wisconsin sites, surface water PFOS concentrations are several orders of magnitude lower than those at the Michigan and Maine sites, and muscle and liver tissue concentrations reflect this pattern accordingly.

Although it is difficult to establish a quantitative relationship between PFOS in surface water and tissue levels, given that ungulates may drink from several water bodies, these findings support the assumption that water is a direct or indirect contributor to PFOS accumulation. The persistence of this water-tissue correlation, despite the influence of other variables such as age, sex, and species, is particularly striking.

### Other potential exposure sources

Liver tissues harvested from elk at the control sites also had detectable levels of PFOS. While these concentrations were lower than the average detected in elk from the former Rocky Flats site, they were still well within the range of concentrations measured at that location. Given that the control sites were not known to be impacted by PFAS spills or releases, these findings suggest that surface water is not the sole driver of PFOS accumulation. The possibility of other exposure pathways cannot be overlooked, considering that PFAS are termed “forever chemicals” and are globally ubiquitous owing to atmospheric deposition [37].

Vegetation consumption may also be an important pathway for PFAS in elk populations. Without exposure to contaminated water, elk in control locations may have been exposed to PFAS by ingesting vegetation. In the current study, we did not analyze the vegetation for PFAS; however native vegetation has been reported to accumulate PFAS [38–40]. Given that other studies did not measure PFAS levels in vegetation nor collect white-tailed deer from a control location, it is unclear how important vegetation is as an exposure pathway and whether this is a consistent pattern among ungulates. Without collecting additional animals from unimpacted sites, we could not draw conclusions regarding PFOS concentrations in herds at control locations.

### Comparison to existing consumption advisories

The PFOS concentrations in white-tailed deer have resulted in the establishment of wildlife consumption advisories. Although wildlife consumption advisories are non-regulatory, they represent an important tool for protecting public health. In Michigan, the detected PFOS levels led to a “do not eat” advisory area for deer obtained within three miles of the marsh adjacent to a U.S. Air Force base. The MDHHS further recommends not eating kidneys or liver from any deer statewide because several chemicals, including PFAS, can accumulate in these organs [29]. Similarly, Maine issued a “do not eat” advisory for deer in an area encompassing multiple farm fields that were reported to contain elevated PFOS levels [30]. Wisconsin issued an advisory recommending that people do not eat livers from deer collected within a five-mile radius of an AFFF manufacturing site [31]. Currently, there are no guidelines for PFAS in wildlife within the State of Colorado, and internal organs are not required to be harvested or prepared as edible meat, although hunters are not prohibited from doing so [41]. Hunting is not permitted at the former Rocky Flats site; however, because elk are not confined to the site, they may still be hunted for food, representing a potential pathway for human exposure to PFAS. However, owing to the small sample size in the current study, it is difficult to draw overall conclusions regarding the human health risks associated with the consumption of elk from the former Rocky Flats site. If additional samples are collected, consumption advisories may be revisited.

### Limitations

The behavior and exposures of animals sampled as a part of this study may or may not represent those living in urbanized areas. Although not specifically discussed in this study, urbanization not only affects the behavior of wildlife but also the number and types of wildlife species present in urban areas. Urban environments reduce certain selective pressures on wildlife while increasing others. For example, pressures on food sources, water and predation may decrease in urban areas, whereas more frequent interactions with humans may increase pressures that alter animal behavior [42].

In the current study, only female elk were used as controls. Female animals are easier to locate because of herd behavior, allowing for easier target selection and sample collection. The control samples provided evidence that PFOS concentrations in the liver tissues of rural animals were similar to those at the former Rocky Flats site. Other results regarding bioaccumulation according to sex and age could not be discerned at the control locations.

**Fig 5:**
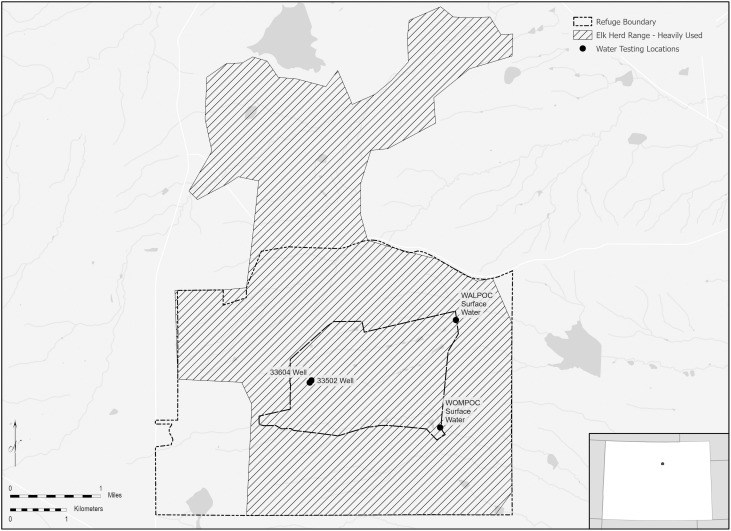
Approximate range of the studied elk subherd shown in relation to USDoE surface water sample locations at the former Rocky Flats site. The map was created using ArcGIS Pro 3.2.3 with National Hydrography Data (NHD) (https://www.usgs.gov/national-hydrography/nhdplus-high-resolution), roads from the National Atlas USGS (http://dds.cr.usgs.gov/pub/data/nationalatlas/roadtrl010g.shp_nt00920.tar.gz), boundary of the national wildlife refuge from the USFWS, and other shapefiles created by Megan E. Klosterman.

Further investigation into the sources of PFAS contamination in the central Front Range area is warranted. GPS collar data indicated that the sampled elk subherd inhabits approximately 5,260 hectares of open space, including nearly 2,100 hectares of the former Rocky Flats site (Fig 5). Of this, less than 240 hectares were affected by industrial activities, with the former fire department comprising less than 1 hectare. The USDoE has documented the historical use of AFFF at the former Rocky Flats site [17]. Given the frequent wildfires in the Front Range of Colorado, firefighting foam has been used in this region for decades. However, ongoing and past industrial activities in the broader area suggest that AFFF is only one of the several potential sources of PFAS contamination.

The transport and fate of PFAS in the environment should be investigated at various scales. This includes determining the background levels of PFAS, given that background levels are likely to vary based on the level of human development. Further research on the fate of PFAS at this site should be conducted. In particular, it would be valuable to determine the importance of soil [43] and herbaceous or woody plants [44] as reservoirs for PFAS.

Finally, further investigations are needed to determine the background concentrations of PFAS in elk residing in the urbanized central Front Range area. The sample size for this study was relatively small, and all animals were collected at a location of known PFAS contamination. Two control animals were sampled from rural Jackson and Saguache Counties, Colorado, located far from reported sources of PFAS contamination. The average PFOS concentrations in liver tissues collected at control locations was 8.75 ± 3.02 ng/g (maximum: 9.40 ng/g) compared with 16.23 ± 4.40 ng/g (maximum: 34.20 ng/g) at the former Rocky Flats site. Although the average concentrations of PFOS in liver tissues were higher at the former Rocky Flats site than in rural Colorado, the data were insufficient to clarify the PFOS patterns among elk in the central Front Range area.

## Supporting information

S1 Table Concentrations of 6:2 FTS in liver and muscle tissues of the 15 elk sampled at the former Rocky Flats site, 2023. (DOCX)

S2 Table Concentrations of 6:2 FTS in liver and muscle tissues of elk at control locations in rural Jackson and Saguache Counties, Colorado, 2023. (DOCX)
