# Supplementary material for: Assessing PFAS exposure in Rocky Mountain elk (Cervus canadensis nelsoni) populations adjacent to the former Rocky Flats nuclear site: A preliminary analysis
Source: PLoS One. 2025 Dec 1;20(12):e0334258. doi: 10.1371/journal.pone.0334258 (PMC12668488; doi:10.1371/journal.pone.0334258)
Supplement: S1 Table — (DOCX) [file pone.0334258.s001.docx]

**S1 Table. Concentrations of 6:2 FTS in liver and muscle tissues of the 15 elk sampled at the former Rocky Flats site, 2023.**

|  | | **6:2 FTS**  **(ng/g)** | |
| --- | --- | --- | --- |
| **Sex** | **Age (years)** | **Liver** | **Muscle** |
| **Female** | **<1** | ND (<0.597) | ND (<0.592) |
|  | **<1** | ND (<12.7) | ND (<0.584) |
|  | **4** | 47.8 | 1.61 (J) |
|  | **5** | 3.07 | 10.4 |
|  | **6** | 34.8 | 1.68 (J) |
|  | **8** | ND (<8.44) | ND (<0.624)  ND (<0.624) ^a^ |
|  | **10** | ND (<0.563) | ND (<0.615) |
|  | **17** | ND (<0.609) | ND (<0.589) |
| **Male** | **<1** | ND (<10.6) | ND (<0.624)  ND (<0.600) ^a^ |
|  | **2** | 39.9 | ND (<0.627) |
|  | **3** | 21.0 | ND (<0.517) |
|  | **3** | 279 (J)  18.9 ^a^ | ND (<0.576) |
|  | **5** | 37.8  44.9 ^a^ | 3.57 |
|  | **7** | 170 | 2.73 |
|  | **7** | 69.8 | ND (<0.606) |

^a^ This table includes duplicate results. Field duplicates and corresponding sample results were used to calculate precision as the relative percent difference (RPD). With the exception of a 3 year-old male animal, which was qualified by the laboratory, all the sample pairs met the desired criteria.
