# Supplementary material for: Assessing PFAS exposure in Rocky Mountain elk (Cervus canadensis nelsoni) populations adjacent to the former Rocky Flats nuclear site: A preliminary analysis
Source: PLoS One. 2025 Dec 1;20(12):e0334258. doi: 10.1371/journal.pone.0334258 (PMC12668488; doi:10.1371/journal.pone.0334258)
Supplement: S2 Table — (DOCX) [file pone.0334258.s002.docx]

**S2 Table. Concentrations of 6:2 FTS in liver and muscle tissues of elk at control locations in rural Jackson and Saguache Counties, Colorado, 2023.**

|  | | **6:2 FTS**  **(ng/g)** | |
| --- | --- | --- | --- |
| **Sex** | **Age (years)** | **Liver** | **Muscle** |
| **Female** | **4** | ND (<9.05)  ND (<7.92) ^a^ | ND (<1.16)  ND (<1.06) ^a^ |
|  | **7** | 30 (J) | 2.42 |

^a^ This table includes duplicate results. Field duplicates and corresponding sample results were used to calculate precision as the relative percent difference (RPD). All the sample pairs met the desired criteria.
